# Super-reflector enabled by non-interleaved spin-momentum-multiplexed metasurface

**DOI:** 10.1038/s41377-023-01118-1

**Published:** 2023-03-24

**Authors:** He-Xiu Xu, Guangwei Hu, Xianghong Kong, Yanzhang Shao, Patrice Genevet, Cheng-Wei Qiu

**Affiliations:** 1 Air and Missile Defense College, Air Force Engineering University, 710051 Xi’an, China; 2 School of Electrical and Electronic Engineering, Nanyang Technological University, Singapore, 639798 Singapore; 3 Department of Electrical and Computer Engineering, National University of Singapore, Singapore, 117583 Singapore; 4 Université Côte d’Azur, CNRS, Centre de Recherche sur l’Hétéro-Epitaxie et ses Applications (CRHEA), 06560 Valbonne, France

**Keywords:** Optoelectronic devices and components, Photonic devices

## Abstract

Electromagnetic wave multiplexing, especially for that occurring at different incidences (spatial-frequency multiplexing), is pivotal for ultrathin multifunctional interfaces and high-capacity information processing and communication. It is yet extremely challenging based on passive and compact wave elements, since the wave excitation and scattering channels are exclusively coupled through gradient phases and hence momentum matching condition at the interface. Here, we propose a spin-momentum multiplexed paradigm called a super-reflector enabling on-demand control of both retroreflections and anomalous reflections using a non-interleaved single-celled metasurface. By multiplexing four channels connecting two spin states excited onto each input of three spatial frequencies, a total of twelve channels are engineered, among which three are retroreflected channels and the residual are anomalous reflection ones. Our compound multiplexed super-reflector allows five degrees of freedom in circular polarization Jones' matrix, approaching the intrinsic upper limit of such planar metasurface. The concept has been experimentally verified by a proof-of-concept super-reflector at microwave frequency, showcasing twelve reflected beams and a high efficiency exceeding 90.6% defined as the ratio of reflected power to incidence for each channel beam. Our strategy opens a new avenue for angle multiplexing and angle-resolved metadevices toward the capacity limit of 2D planar Jones’ matrix.

## Abstract (web summary)

A super-reflector was reported to enable on-demand control of both retroreflections and reflections of circularly polarized wave. Specifically, it performs four-channel abnormal reflections at each incidence of three multiplexed spatial frequencies.

## Graphical abstract

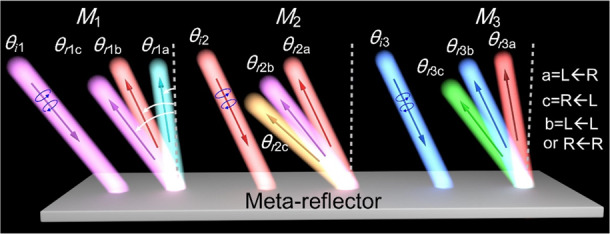

## Introduction

Retroreflector supports the reflection of electromagnetic (EM) wave back to where it comes from even at oblique incidence. Such peculiar reflective behaviors of waves carrying the information and energy are important for applications including laser tracking, target labeling, navigation safety, radar cross-section/visibility enhancement, remote sensing, satellite communication and others. Over the decades, the corner reflector and Luneburg lens have been reported^1–3^ via assembling different optical materials or elements for retroreflections. This however renders the bulk size, large weight and nonplanar configuration, hindering the real-world applications where the integration, miniaturization, and compatibility of existing wave systems are favored.

**Fig. 1:**
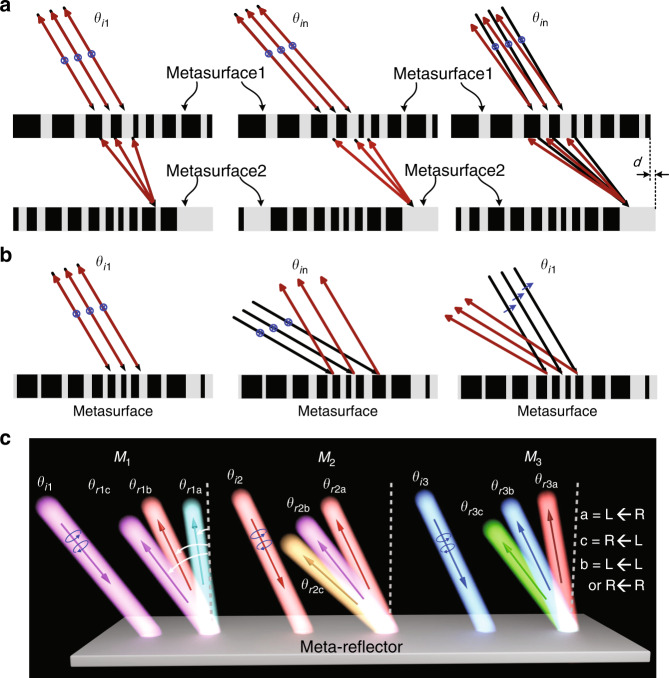
Evolution of available of retroreflectors with their advantages and disadvantages. **a** Dual-metasurface retroreflector enabling backward reflection at various incident angles (left and middle panel), while relying on highly precise alignment (right panel)^10^. **b** Typical single-metasurface retroreflector only for one incidence (left and middle panel) and one polarization channel (right panel). **c** Angle-multiplexed super meta-reflector for synergized reflections and retroreflections at three excitation momentums of *M*_1_, *M*_2_, and *M*_3_, advancing a big step relative to previously reported ones only for retroreflection. The wave is incident along three different angles of *θ*_i1_, *θ*_i2_, and *θ*_i3_, four reflections with three different angles of *θ*_ra_, *θ*_rb_, and *θ*_rc_ (*θ*_ra_ < *θ*_rb_ < *θ*_rc_) are expected along four CP channels at each incidence. Here, the subscripts ‘a’ and ‘c’ indicate the channel of LR (RCP to LCP) and RL (LCP to RCP), respectively, while ‘b’ represents the channel of LL (LCP to LCP) or RR (RCP to RCP)

Recently, there are growing trends to explore the metasurface, a planar structure consisting of artificially engineered subwavelength meta-atoms, for flat and compact electromagnetic devices to offer complete control the amplitude, phase, and polarization (APP) of EM wave or light^4–9^ Employing metasurfaces for retroreflectors has been an intriguing topic most recently^10–17^ In the seminal work^10^, dual metasurfaces are utilized, the first of which performs a spatial Fourier transform and its inverse while the other imparts a spatially varying momentum to the Fourier transform. Although retroreflections can be engineered at various incidences, the slight misalignment of dual plates will significantly undermine the efficiency and even dissolve the retroreflection, as shown in Fig. 1a. Single-metasurface retroreflectors well release the issue of stringent alignment^11–17^ and even enables adaptability via mechanically altering the geometry of reconfigurable C-shaped resonators^13^, switchable metagrating^14^, and double C-shaped meta-atoms^16^. Nevertheless, those demonstrations are particularly designed for one specific spatial frequency and polarization channel, see Fig. 1b. To date, the high-capacity retroreflector particularly with multichannel retroreflections occurring at different spatial frequencies^18^ is still extremely challenging and remained unexplored, considerably hindering the real-world applications in practice. As to the multitasking metasurfaces for high-capacity and multiple functionalities, various approaches with frequency, angular, polarization, orbital angular momentum, or spatial multiplexing have been explored^19–40^, such as interleaved nanostructures in basic element, supercell and even global metasurface, stacking them in multilayers, and other design of meta-atoms. Particularly, diatomic^41,42^ and even tetratomic^43–45^ meta-atom designs were reported with maximal degrees of freedom (DoFs) of fourfold and sixfold multitasking purpose. Nevertheless, the channel interference or crosstalk therein is inevitable due to inter-element coupling, which significantly limits the efficiency. To circumvent this challenge, a single-celled metasurface, instead of a multilayer or interleaving approach, was reported for complete wave manipulation via orientation degeneracy^46,47^ However, very limited levels of discretized dynamic and geometric phases were attainable, leading to large phase tolerances and thus compromise of the overall performance of resulting devices.

Here, we report a novel concept of super-reflector, which enables on-demand control of both retroreflections and anomalous reflections of circularly polarized (CP) wave using non-interleaved single-celled metasurface. It affords a novel avenue for multi-angle operations that was previously only attainable in the dual-metasurface scheme, but completely eliminates the drawbacks of stringent alignment in dual-metasurface and monotonous single-channel backscattering in one metasurface, going beyond available strategies^10–17^ Specifically, it performs four-channel abnormal reflections at each incident angle of three multiplexed different spatial frequencies, see Fig. 1c. As a result, an overall of twelve full co-polarized and cross-polarized CP channels are engineered for high capacity by switching CP states of excitations and detections. We emphasize that the coalescence of anomalous reflection and retroreflection explored here is intrinsically general, leveraging the functionalities of all previously reported individual reflection or retroreflection. Moreover, the effect of inter-channel crosstalk is here judiciously eliminated by the full two-dimensional (2D) parametric mapping. Our strategy for multichannel retroreflections by combining spin-multiplexing and spatial-frequency (momentum) multiplexing is only attainable by coherently synergizing dynamic phase and decoupled geometric phase, and is thus completely different from controlling angular dispersions^48^.

## Results

### Principle for synergized reflection and retroreflection

The principle behind proposed angle-multiplexed reflections and retroreflections is to impose three phase gradients for triple retroreflections into one metasurface, i.e., each gradient is imparted under one specific CP–CP channel of four at each tilt angle of three, respectively. Then the gradients at other helicity channels are inherently deterministic with specified reflection angles due to the commonly fixed relationship of dynamic and geometric phase among four co- and cross-CP channels. Since the concept of super-reflector is connected with spin multiplexing and spatial-frequency multiplexing, here we discuss the principle by starting from a general CP Jones’ matrix $$R_{CP} = \left[ {\begin{array}{*{20}{c}} {\left| {r_{LL}} \right|e^{\varphi _{LL}}} & {\left| {r_{RL}} \right|e^{\varphi _{RL}}} \\ {\left| {r_{LR}} \right|e^{\varphi _{LR}}} & {\left| {r_{RR}} \right|e^{\varphi _{RR}}} \end{array}} \right]$$ of planar 2D structures in a reflective scheme, where the subscripts *L* and *R* denote left- and right-handed wave, respectively. Here, $\varphi _{LL}/\left| {r_{LL}} \right|$, $\varphi _{RL}/\left| {r_{RL}} \right|$, $\varphi _{LR}/\left| {r_{LR}} \right|$, and $\varphi _{RR}/\left| {r_{RR}} \right|$ are phase/amplitude of four LCP/RCP reflection components under LCP/RCP wave excitation. Although eight components are available, only six of |*r*_RL_|/|*r*_LR_|, |*r*_LL_|/|*r*_RR_|, *φ*_LR_, *φ*_RL_, *φ*_RR_, and *φ*_LL_ are identified as the upper limit of DoFs due to the bound of reciprocity and lossless conditions. *φ*_LR_ and *φ*_RL_ can be decoupled by imposing different phases on co- or cross-polarized linearly polarized (LP) channels along its fast (*x*) and slow (*y*) axes rotated by an angle *Φ*^49^. To elucidate the possibility of independent control, we write those components in the linearly polarized (LP) basis as

1a $$r_{LR}{{{\mathrm{ = }}}}\frac{1}{2}\left[ {\left( {r_{xx} - r_{yy}} \right) - j\left( {r_{xy} + r_{yx}} \right)} \right]{{{\mathrm{e}}}}^{ - j2\Phi }$$

1b $$r_{RL}{{{\mathrm{ = }}}}\frac{1}{2}\left[ {\left( {r_{xx} - r_{yy}} \right) + j\left( {r_{xy} + r_{yx}} \right)} \right]{{{\mathrm{e}}}}^{ + j2\Phi }$$

1c $$r_{LL}{{{\mathrm{ = }}}}\frac{1}{2}\left[ {\left( {r_{xx} + r_{yy}} \right) + j\left( {r_{xy} - r_{yx}} \right)} \right]$$

1d $$r_{RR}{{{\mathrm{ = }}}}\frac{1}{2}\left[ {\left( {r_{xx} + r_{yy}} \right) - j\left( {r_{xy} - r_{yx}} \right)} \right]$$

Here, $\varphi _{xx}/\left| {r_{xx}} \right|$, $\varphi _{yx}/\left| {r_{yx}} \right|$, $\varphi _{xy}/\left| {r_{xy}} \right|$, and $\varphi _{yy}/\left| {r_{yy}} \right|$ are dynamic phase/amplitude of four *x*/*y*-polarized reflection components under *x*/*y* polarization. For a co-polarized system with mirror symmetry, it is readily to realize specific co- and cross-polarized LP components fulfilling $\left| {r_{yx}} \right| \approx \left| {r_{xy}} \right| \approx 0$ and $\left| {r_{xx}} \right| \approx \left| {r_{yy}} \right| \approx 1$. In that case, Eq. (1) is immediately simplified as

2a $$r_{LR} = \sin \frac{{\varphi _{xx} - \varphi _{yy}}}{2}{{{\mathrm{e}}}}^{ - j\left( {\frac{{\varphi _{xx} + \varphi _{yy}}}{2} + 2\Phi } \right)}$$

2b $$r_{RL} = \sin \frac{{\varphi _{xx} - \varphi _{yy}}}{2}{{{\mathrm{e}}}}^{ - j\left( {\frac{{\varphi _{xx} + \varphi _{yy}}}{2} - 2\Phi } \right)}$$

2c $$r_{LL} = \cos \frac{{\varphi _{xx} - \varphi _{yy}}}{2}{{{\mathrm{e}}}}^{ - j\left( {\frac{{\varphi _{xx} + \varphi _{yy}}}{2}} \right)}$$

2d $$r_{RR} = \cos \frac{{\varphi _{xx} - \varphi _{yy}}}{2}{{{\mathrm{e}}}}^{ - j\left( {\frac{{\varphi _{xx} + \varphi _{yy}}}{2}} \right)}$$

Equation (2) reveal that two levels of amplitude profile (|*r*_RL_|=|*r*_LR_|, and |*r*_LL_|=|*r*_RR_|) and three types of phase pattern (*φ*_LR_, *φ*_RL_, and *φ*_RR_/*φ*_LL_) can be engineered individually provided a rotation and an appropriate phase difference between *φ*_xx_ and *φ*_yy_. For example, a maximum of four channels can be engineered with uniform intensity by setting *φ*_xx_–*φ*_yy_ = π/2, while polarization channels are extremely reduced if we want to keep (*φ*_xx_–*φ*_yy_ = 0) or switch (*φ*_xx_–*φ*_yy_ = π) the output helicity with high efficiency for some particular applications. Besides, the intensity of different reflection channels can be arbitrarily controlled using other values of phase difference. As shown in Supplementary Fig. S1, the realized five DoFs is approaching an upper limit of six in a CP Jones’ matrix of reciprocal 2D single-celled metasurface, and advances a significant step towards ultimate controls of light in relative to tetratomic scheme^43–45^ Note that co-polarized transmissive phases *φ*_LL_ and *φ*_RR_ have been decoupled by introducing chirality as a new DoF;^49^ however, the strategy of reversely rotating top and bottom patterns are not feasible for a reflective scheme due to mirror operation of a metallic ground. In the following, we will utilize those maximum five DoFs for spin multiplexing, and further combine momentum multiplexing to implement multifunctional retroflections and reflections.

By definition, the design of retroreflector requires inversion of the light tangential momentum in the transverse plane. To flip the in-plane component of the momentum of incident wave (***p***_||_) at wavelength *λ*, we need to impart an exact momentum (***p***_m_=−2***p***_||_) on the gradient metasurface for compensation, yielding $\sin \theta _i - \sin \theta _r = 2\sin \theta _i = \frac{\xi }{{k_0p}} = \frac{\lambda }{\Gamma }$ for momentum conservations. Therefore, the required phase gradient of the angle-multiplexed retroreflector at *θ*_i_ should fulfill $\xi = 2k_0p\sin \theta _i$. Here, *i* and *r* denote the incidence and reflection, respectively, *k*_0_ is the wavevector in free space, *p* is the lattice constant and *Γ* is length of the supercell. For tri-channel retroreflections at *θ*_i1_, *θ*_i2_ and *θ*_i3_, we impose the required phase gradients (*ξ*_RL_, *ξ*_LR_, *ξ*_LL_/*ξ*_RR_) to four different CP channels of RL, LR and LL/RR, respectively at each *θ*_i_. Then, we immediately achieve the following formalisms as a function of dynamic phase (${\xi}_{0} = ( {\varphi _{xx} + \varphi _{yy}} )/2$) and geometric phase (2Φ) gradient according to Eq. (2).

3a $$2k_0p\sin \theta _{i1} = \xi _{RL} = \xi _0 + 2\Phi$$

3b $$2k_0p\sin \theta _{i2} = \xi _{LR} = \xi _0 - 2\Phi$$

3c $$2k_0p\sin \theta _{i3} = \xi _{LL} = \xi _{RR} = \xi _0$$

Here, Φ is associated with the orientation angle of each meta-atom, which provides the geometric phase at the interface^50^. At each *θ*_iN_, there are three open diffraction channels with reflection angles occurring at *θ*_rNa_, *θ*_rNb_, and *θ*_rNc_ (*θ*_rNa_ < *θ*_rNb_ < *θ*_rNc_) along full CP channels of LR, LL/RR, and RL. Then, the corresponding angles of *θ*_rNa_, *θ*_rNb_ and *θ*_rNc_ can be exclusively calculated based on the following established 12 equations.

4a $$k_0p\left( {\sin \theta _{iN} + \sin \theta _{rNa}} \right) = \xi _{LR} = \xi _0 - 2\Phi$$

4b $$k_0p\left( {\sin \theta _{iN} + \sin \theta _{rNb}} \right) = \xi _{LL} = \xi _{RR} = \xi _0$$

4c $$k_0p\left( {\sin \theta _{iN} + \sin \theta _{rNc}} \right) = \xi _{RL} = \xi _0 + 2\Phi$$

Here, *N* denotes the N^*th*^ excitation momentum and is chosen as 1, 2, and 3 for spatial-frequency multiplexing. By combining Eqs. (3) and (4), it is concluded that $\theta _{r1c} = \theta _{r2b} = \theta _{i1}$, $\theta _{r2a} = \theta _{r1b} = \theta _{i2}$, and $\theta _{r3b} = \theta _{i3}$. As a result, a total of twelve beams, within which seven are directed toward distinct diffraction angles, will be formed in backward scattering channels, among them three as retroflections and four as anomalous reflections. The angles of the remaining five channels can be calculated as

5a $$\theta _{r1a} = \arcsin \left( {2\sin \theta _{i2} - \sin \theta _{i1}} \right)$$

5b $$\theta _{r2c} = \arcsin \left( {2\sin \theta _{i1} - \sin \theta _{i2}} \right)$$

5c $$\theta _{r3a} = \arcsin \left( {\frac{{3\sin \theta _{i2} - \sin \theta _{i1}}}{2}} \right)$$

5d $$\theta _{r3b} = \arcsin \left( {\frac{{\sin \theta _{i2} + \sin \theta _{i1}}}{2}} \right)$$

5e $$\theta _{r3c} = \arcsin \left( {\frac{{3\sin \theta _{i1} - \sin \theta _{i2}}}{2}} \right)$$

### Meta-atom and metasurface design

**Fig. 2:**
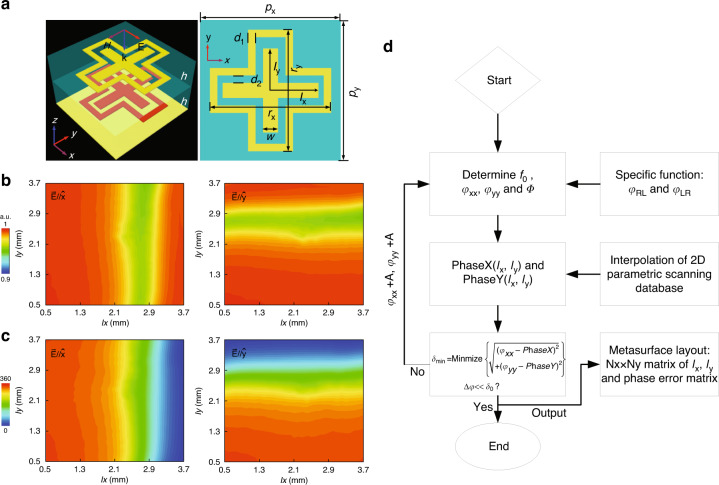
Design of meta-atom and resulting metasurface. **a** Layout of the meta-atom. Numerically calculated **b** amplitude (|*r*_xx_| and |*r*_yy_|) and **c** phase (*φ*_xx_ and *φ*_yy_) database at 10 GHz under *x*- and *y*-polarized wave. The residual geometric parameters for top dual-layer element are detailed as: *p*_x_ = *p*_y_ = 11 mm, *r*_x_ = *r*_y_ = 9 mm, *d*_1_ = *d*_2_ = 0.5 mm, and *w* = 1 mm. **d** The CAD process of determining metasurface layout. Here, *A* is an additional variable that can be gradually changed as required. δmin is the minimum distance characterized by the least square algorithm

With the approach established above, it is ready to theoretically predict the required 2D *φ*_xx_, *φ*_yy_ and Φ distributions across the metasurface for such purpose. We now conceive an anisotropic co-polarized meta-atom to realize above specified *φ*_xx_, *φ*_yy_, and Φ. Figure 2 shows the geometry and corresponding EM response of the basic building block. As illustrated in Fig. 2a, the meta-atom is a dual-layer composite metallic pattern, which is specifically designed to contain an inner cross patch and an external cross loop to afford two resonance modes to fulfill 2π phase coverage when excited with two orthogonal LP waves. The lattice constant is designed as 11 × 11 mm^2^ to target the center frequency at 10 GHz. Here, we performed a 2D parametric scanning of *l*_x_ and *l*_y_ within 0.5–3.7 mm in steps of 0.1 mm while keeping other geometric parameters constant. We obtain reflection response database of each meta-atom for precise phase mapping. As shown in Fig. 2b, a gap (as the resonant dip) is observed across the amplitude spectrum in both polarization states. Moreover, the intensity |*r*_xx_| and |*r*_yy_| is larger than 0.95 for all *l*_x_ and *l*_y_, which facilitates the high-efficiency phase-only design. As shown in Fig. 2c, the phase *φ*_xx_ and *φ*_yy_ decreases progressively as *l*_x_ and *l*_y_ increase, respectively, indicating that *l*_x_ dominantly determines *φ*_xx_ while *φ*_yy_ is mainly dependent on *l*_y_. The phase coverage is observed about 332º at 10 GHz. Most importantly, the minor fluctuation is observed in both amplitude and phase map, i.e., uneven |*r*_xx_| and *φ*_xx_ curve at any *l*_x_ when it is varied along *l*_y_, vice versa for |*r*_yy_| and *φ*_yy_, indicating a non-negligible polarization crosstalk. Such a phase fluctuation makes the one-dimensional (1D) mapping of *φ*_*xx*_ ~ *l*_x_ and *φ*_*yy*_ ~ *l*_y_ is unfeasible for high-efficiency designs. To minimize the crosstalk, a computer-aided design (CAD) process is developed for optimum metasurface layout based on above 2D mapping (PhaseX ~ (*l*_x_, *l*_y_) and PhaseY ~ (*l*_x_, *l*_y_)), see Fig. 2d and more details in the Experimental Section. Such a 2D mapping process can minimize the phase tolerances induced by polarization crosstalk, advancing an enormous step relative to previous one-dimensional mapping^35^. To sum up, the metasurface layout can be fixed by determining *l*_x_ and *l*_y_ of each meta-atom according to *φ*_*xx*_ and *φ*_*yy*_ distributions while figuring out the orientations of each meta-atom by a rotation of Φ.

### Spin-multiplexed multi-beam generator

**Fig. 3:**
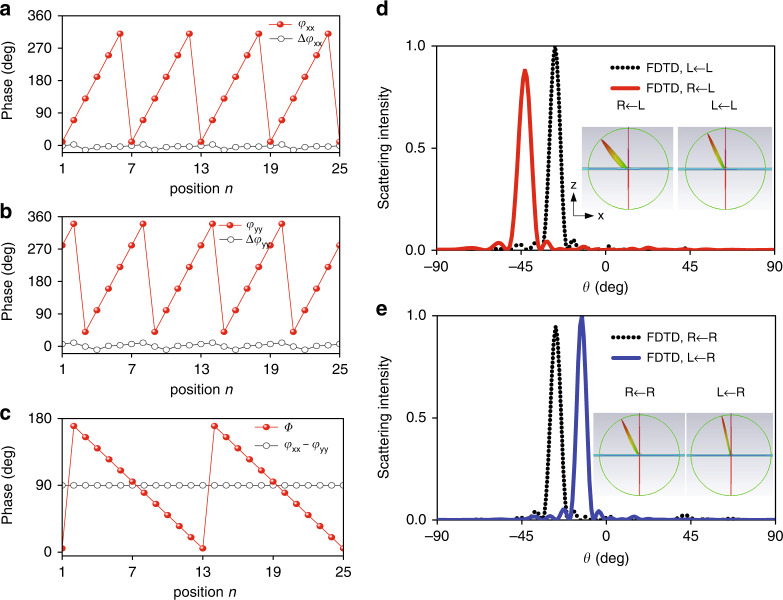
FDTD characterization of spin-multiplexed quad-channel multi-beam generator. Required birefringent dynamic phases **a** *φ*_*xx*_ and **b** *φ*_*yy*_ of finally utilized meta-atoms, and practical near-zero phase tolerance profile of Δ*φ*_*xx*_ and Δ*φ*_*yy*_ under **a** *x*- and **b** *y*-polarized wave, respectively. Here, Δ*φ*_*xx*_ and Δ*φ*_*yy*_ are deviations between theoretically calculated phases and those of practically utilized meta-atoms, indicating negligible phase errors. **c** Required half geometric phase, and phase difference between *φ*_*xx*_ and *φ*_*yy*_. FDTD calculated 3D and 2D cross-section scattering patterns at 10 GHz impinged by **d** LCP and **e** RCP wave, respectively

Before verifying the compound spin-and-momentum multiplexing concept in reflection scheme, we first demonstrate the spin multiplexing by engineering a quad-channel multi-beam generator composed of 25 × 25 meta-atoms, which can be considered a special case of Eq. (4) by setting *n* = 1 and *θ*_*I*_ = 0. The beams are steered along x axis via independent control of five DoFs in planar 2D CP Jones matrix. Two predicted phase gradients of $\xi _{RL} = k_0p\sin \theta _{r1} = \pi /2$ and $\xi _{LR} = k_0p\sin \theta _{r2} = \pi /6$ are imparted into RL and LR channels. Consequently, the third beam is directed toward $\theta _{r3} = \arcsin \left( {\sin \theta _{r1}/2 + \sin \theta _{r2}/2} \right)$ along LL or RR channel. To implement desired uniform intensity of |*r*_RL_|=|*r*_LR_|=|*r*_LL_|=|*r*_RR_| among four CP channels, the birefringent dynamic phase deviation of each meta-atom fulfills *φ*_xx_ – *φ*_yy_ = π/2. Then, the finally required birefringent dynamic phases and half geometric phase for synthesizing full CP channels are given as $\varphi _{xx} = \frac{1}{2}\left( {\varphi _{RL} + \varphi _{LR}} \right)$, $\varphi _{yy} = \frac{1}{2}\left( {\varphi _{RL} + \varphi _{LR} - \pi } \right)$, and $\Phi = \frac{1}{4}\left( {\varphi _{RL} - \varphi _{LR}} \right)$, see Fig. 3a–c. Based on these phase profiles, the meta-sheet layout along *x* axis as depicted in Supplementary Fig. S2 is determined based on the developed 2D CAD mapping process and established 2D scanning reflection database.

As is shown, the phase errors are within ${-12.5-9.6^{\circ}}$for both Δ*φ*_*xx*_ and Δ*φ*_*yy*_, which is reasonably small for a precise and high-efficiency design. Figure 3e and f plot the FDTD calculated 3D and 2D cross-section scattering patterns of the meta-sheet impinged by LCP and RCP wave, respectively. As expected, four anomalous beams are observed along LL, RL, RR, and LR channel, respectively at 10 GHz, with two of which observed at the same angle. These beams are directed toward *θ*_rc_ = −43°, *θ*_rb_ = −27° and *θ*_ra_ = −13° with almost identical intensity, coinciding well with the theoretically calculated ones at *θ*_rc_ = −42.6°, *θ*_rb_ = −27°, and *θ*_ra_ = −13.1°. The widened beam intrinsically gives rise to the relatively low peak value at a large scanning angle. In all cases, pure single-mode operation is observed, where other specular and higher-order reflection modes are mostly suppressed, corresponding to a high efficiency of near 100%. More importantly, the spin-multiplexing strategy proposed here can be readily extended to engineer other large-capacity metadevices such as lenses and axicons with multiple versatile foci, and non-diffractive propagation length, and also vortex-beam generator with different topological charges.

### Spin-momentum multiplexed super-reflector

Provided full four-channel spin multiplexing, we now further proceed to design a more complicated spin-momentum multiplexed super-reflector which can be considered as a super integration of triple retroreflectors and four anomalous reflectors. It is targeted at 10 GHz and is also composed of 25 × 25 meta-atoms. Here, two retroreflection angles are predesigned as *θ*_i1_ = −30º and *θ*_i2_ = −15º. Then the third retroreflection angle is inherently determined as *θ*_i3_ = −22.3º according to the principle derived in Section “Principle for synergized reflection and retroreflection”. Note that the retroreflections can be engineered at any three different incident angles instead of two arbitrary ones here provided an additional DoF of chirality for completely decoupled *φ*_LL_ and *φ*_RR_. Moreover, theoretically, there is no limitation to refletroflection angles of *θ*_i1_ and *θ*_i2_, which can be arbitrarily selected provided that the maximum output angle *θ*_r2c_ does not exceed −90º. In that case, a surface wave is supported other than a propagating wave for a spin-momentum multiplexed retroreflector.

**Fig. 4:**
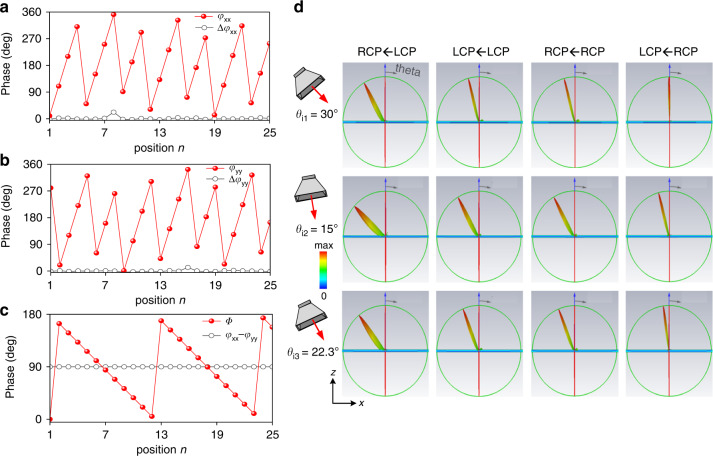
FDTD characterization of the spin-and-momentum multiplexed retroreflector. Synthesized birefringent dynamic phases *φ*_*xx*_ and **b** *φ*_*yy*_ of finally utilized meta-atoms, and practical near-zero phase tolerance profile of Δ*φ*_*xx*_ and Δ*φ*_*yy*_ under **a** *x*- and **b** *y*-polarized wave, respectively. Here, Δ*φ*_*xx*_ and Δ*φ*_*yy*_ are deviations between theoretically calculated phases and those of practically utilized meta-atoms, indicating negligible phase errors. **c** Required half geometric phase, and phase difference between *φ*_*xx*_ and *φ*_*yy*_. **d** FDTD calculated 3D scattering patterns under three different incidences at four channels by altering orthogonal CP states of incident and output wave

Figure 4a–c shows the theoretically calculated phase patterns of *φ*_*xx*_, *φ*_*yy*_, and *Φ* according to the principle of generalized reflection and retroreflection. Using a similar CAD approach in the Experimental Section, we retrieved the required *l*_x_ and *l*_y_ as shown in Supplementary Fig. S3. Again, the phase tolerance of each meta-atom is quite small, which is within −2.8–22.5° for *φ*_*xx*_ and −3.9–12.3° for *φ*_*yy*_. Figure 4d illustrates the calculated scattering patterns of the designed retroreflector via FDTD method. As is expected, the angles of output beams are varied at three angles of incidence when CP states of incident and output wave are flipped, and the scattering intensity of four beams is maintained almost the same. Specifically, at *θ*_i1_ = −30°, the wave propagates back (*θ*_r1c_) at RL channel, while is directed toward *θ*_r1b_ = −15° at RR/LL channel and to *θ*_r1a_ = −1° at LR channel. At *θ*_i2_ = −15°, the wave is reflected back (*θ*_r2a_) at LR channel, and steered to *θ*_r2b_ = −30° at RR/LL channel and to *θ*_r2c_ = −47.8° at RL channel. Instead at *θ*_i3_ = −22.3°, the beam (*θ*_r3b_) is backward at RR/LL channel and appears at *θ*_r3c_ = 38.3° and *θ*_r3a_ = 7.9º in RL and LR channel, respectively. The beam directions in all cases coincide well with the theoretically calculated ones. In all, seven far-field scattering beams are engineered with distinct tailored directions, among which three are retroreflections and four are abnormal reflections. Most importantly, the single-mode operation with other modes almost suppressed indicates a high efficiency of almost 100%.

**Fig. 5:**
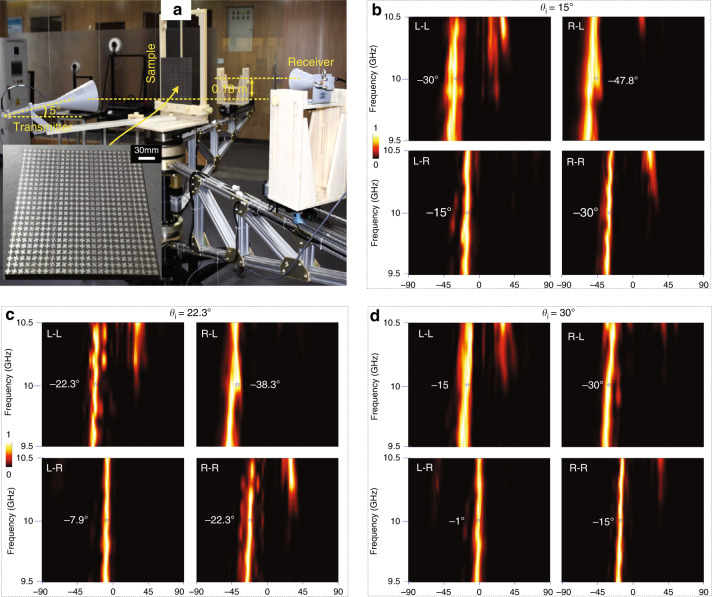
Experimental characterization of the spin-and-momentum multiplexed retroreflector under three angles of incidence. **a** Far-field scattering measurement setup using a 2D rotary table. Scattering intensity map (|*E*|^2) of the retroreflector from 9.5 to 10.5 GHz within −90–90° in steps of 1° at **b** *θ*_i2_ = −15°, **c** *θ*_i3_ = −22.3° and **d** *θ*_i1_ = −30° by switching the handedness of CP input and output. All patterns are normalized to their maximum. Here, the theoretically calculated beam angle is indicated by blue star

For verifications, the dual-layer proof-of-concept sample was experimentally characterized, see measurement setup and photograph of fabricated prototype shown in Fig. 5a. The triple-layer metallic patterns of each metadevices were fabricated individually on two dielectric boards using PCB technique and then assembled through adhesives. The sample was characterized using an automatically 2D rotary table, where a received horn antenna was fixed on an automatically rotated arm around the sample within −90–90° in steps of 1°. More experimental details can be referred to the Experimental Section. Figure 5b–d plots the scattering intensity map (|*E|*^2) of the retroreflector from 9.5 to 10.5 GHz under three angles of incidence. As can be seen, the measured scattering beams indicated by light red flow covers exactly the theoretically calculated one denoted by blue star at 10 GHz in all cases, verifying rationality of our design. The measured angle error is observed within ±0.5º. Moreover, undesired specular and high-order reflection modes occur at low edge frequencies for co-CP channels while are almost completely eliminated at all observed frequencies within 9.5–10.5 GHz for cross-CP components. This is because the cross-CP components in LR and RL channels are mainly determined by the dispersionless geometric phases. In sharp contrast, the co-CP counterparts in LL and RR channels are dominated by the dispersive dynamic phases, and a specifically engineered meta-atom with low-quality factor would improve the robustness of the design against frequency. Nevertheless, the single scattering mode is again inspected at target frequency 10 GHz for all measured CP channels. Such a proposal finds strong support from the cross-section pattern shown in Supplementary Fig. S4, whereas the FDTD calculations are in good consistency with the experiments. The lowest operation efficiency is measured as 90.6% at LL channel under −15º tilt angle incidence.

## Discussion

To summarize, we have proposed and experimentally demonstrated a high-capacity non-interleaved metasurface paradigm which multiplexes full polarization channels excited at three input spatial frequencies. By synergizing geometric phase and dynamic phase for CP-channel decoupling, and by encoding three tailored phase patterns to decoupled CP channels according to momentum conservation at three incidences, twelve-channel abnormal beams are realized, among which three are retroreflections and the other nine are anomalous reflections. A super-reflecting metasurface prototype was experimentally demonstrated at microwave frequency by altering the CP states of input and output ends. The efficiency is measured more than 90.6% for all twelve channels under three different incident angles, which is harder to achieve compared with previous devices. This is especially valuable for the retroreflection, and such an efficiency indicates a highest one among available data. More independent retroreflections and reflections can be engineered at a maximum of sixteen channels at four incidence angles by completely decoupling *φ*_LL_ and *φ*_RR_, which is a further generalization of current approach. Our angle-multiplexing approach is deterministic, and sets a solid platform for large-capacity, angle-resolved and multi-target tracking applications in a compact footprint.

## Materials and methods

### CAD design

For an easy layout mapping, a CAD process is established which can automatically construct all metallic patterns through program codes in a commercial software based on 1D or 2D reflection response database. Specifically, the mapping of top dual-layer composite metasurface layout is performed based on least square algorithm and the 2D scanning phase database PhaseX and PhaseY, see Fig. 2d. The design accuracy of 2D structural parameters *l*_x_ and *l*_y_ is guaranteed by minimizing the sum of squares of phase errors between the ideal phase matrices $\varphi _{xx}\left( {x,y} \right)$ and $\varphi _{yy}\left( {x,y} \right)$ and scanning phase matrices PhaseX and PhaseY according to the minimum error function $\delta _{\min }$.

### Numerical characterizations

All numerical designs and FDTD characterizations are performed through numerical simulation package CST Microwave Studio. In calculations of the reflection amplitudes/phases of the meta-atom, especially in generating the reflection response database, we impose periodic boundary conditions at its four bounds, and with a Floquet port placed at a distance 15 mm away from the meta-atom plane. In calculating the scattering behavior of multi-beam generator and angle-multiplexed retroreflector, open condition is set to the ends of the inhomogeneous array while periodic boundary is assigned along the uniform direction to save computing resources.

### Microwave experiments

The far-field scattering experiments is carried out using a 2D rotary table, see Fig. 5a. In experiments, the transmitted horn was fixed to a plate, while the received horn was fixed on an automatically rotated arm, which was controlled by an electronic motor to move around the sample in a circumference. To avoid normal blocking of any signal, the transmitting horn is deliberately tilted up 5º and is placed below the received one by 0.18 m. Moreover, to afford a quick plane-wave excitation without much divergence of antenna fields, we utilize a broadband dual-CP dielectric lens horn antenna with an aperture of 280 mm and an axial ratio of less than 3.5 dB within 6–18 GHz. A small-sized broadband dual-CP horn with an aperture of 75 mm and an axial ratio of less than 3.5 dB within 8–18 GHz is utilized as the receiver. It received signals in a circumference in steps of 1º.

## Supplementary information

Super-Reflector Enabled by Non-Interleaved Spin-Momentum-Multiplexed Metasurface
