# Supplementary material for: Super-reflector enabled by non-interleaved spin-momentum-multiplexed metasurface
Source: Light Sci Appl. 2023 Mar 24;12:78. doi: 10.1038/s41377-023-01118-1 (PMC10039034; doi:10.1038/s41377-023-01118-1)
Supplement: Supplementary file 1 — Super-Reflector Enabled by Non-Interleaved Spin-Momentum-Multiplexed Metasurface [file 41377_2023_1118_MOESM1_ESM.docx]

Supplementary Information for

**Super-Reflector Enabled by Non-Interleaved Spin-Momentum-Multiplexed Metasurface**

*He-Xiu Xu^1^*^*^*, Guangwei Hu^2^, Xianghong Kong^3^, Yanzhang Shao^1^, Patrice Genevet^4^, Cheng-Wei Qiu^3*^*

*^1^Air and Missile Defense College, Air Force Engineering University, Xi'an 710051, China*

*^2^School of Electrical and Electronic Engineering, Nanyang Technological University, Singapore 639798, Singapore*

*^3^Department of Electrical and Computer Engineering, National University of Singapore, Singapore 117583, Singapore*

*^4^Université Côte d’Azur, CNRS, Centre de Recherche sur l’Hétéro-Epitaxie et ses Applications (CRHEA), 06560 Valbonne, France*

*Corresponding Authors: He-Xiu Xu (hxxuellen@gmail.com); Cheng-Wei Qiu (eleqc@nus.edu.sg)

**Keywords:** Retroreflection; anomalous reflection; decoupled metasurface; momentum multiplexing; spin multiplexing

1. **Illustration of the degree of freedom for wave control**

Our reported triplex angle channels are only attainable by coherently synergizing dynamic phase (DP) and decoupled geometric (PB) phase shown in Figure S1, where five DoFs of |*r*_RL_|/|*r*_LR_|, |*r*_LL_|/|*r*_RR_|, *φ*_LR_, *φ*_RL_ and *φ*_RR_/*φ*_LL_ approaching an upper limit of six in a CP Jones’ matrix of reciprocal planar two-dimensional (2D) structures are realized, which is a significant step towards ultimate controls of light in such single-celled structures.


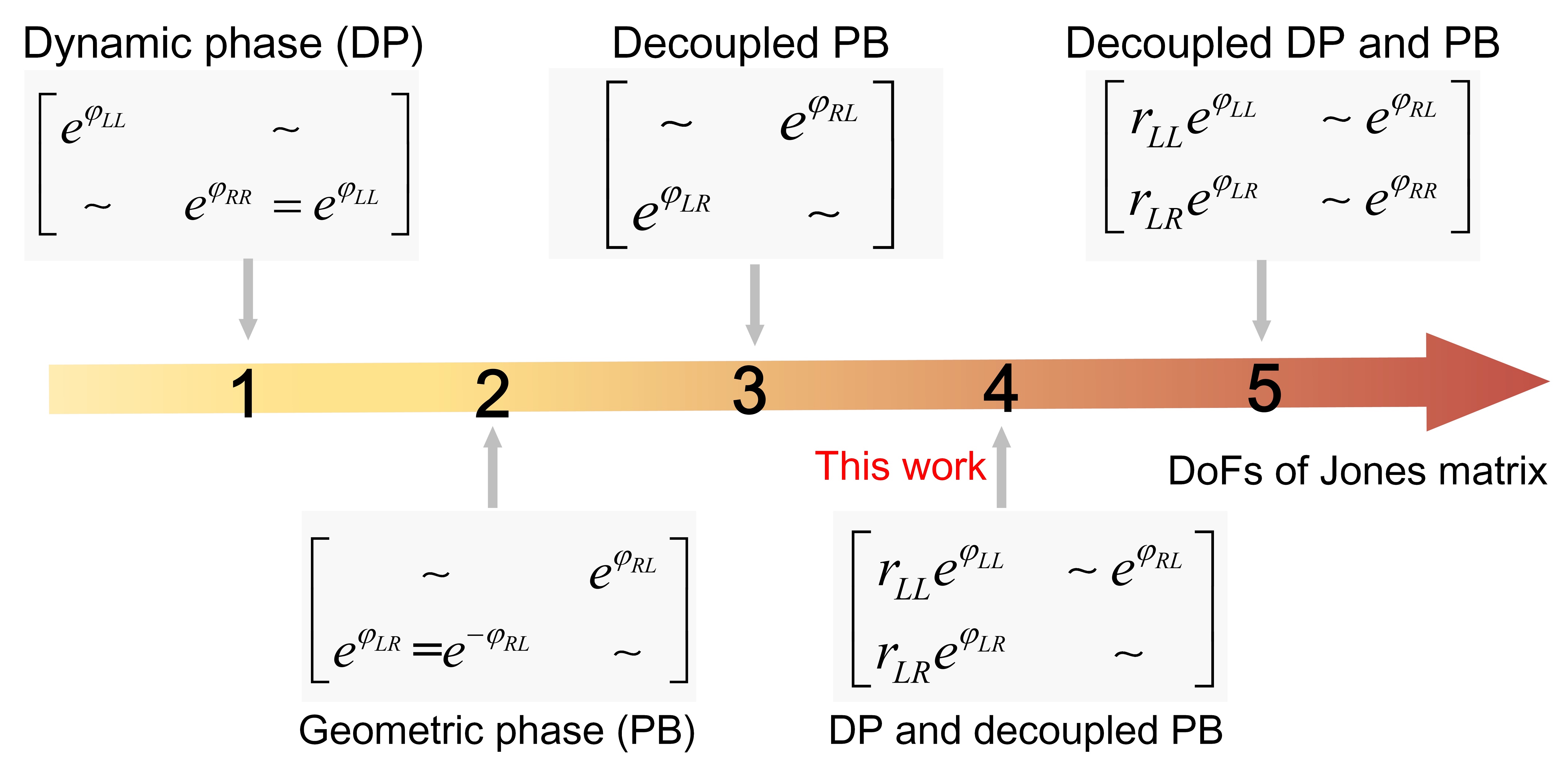


**Figure S1** Illustration of utilized number of DoFs of the CP Jones matrix, here the arrow indicates the development trend along more DoFs.

1. **Detailed structure layout for multi-beam generator**

Figure S2 portrays the final meta-sheet layout based on the retrieved key structure parameters *l*_x_ and *l*_y_, which is determined based on 2D scanning reflection database and our established 2D CAD mapping process. As is shown, the orientation along with structure parameters *l*_x_ and *l*_y_ vary point by point. The change of *l*_x_ is kept synchronously with that of *l*_y_, guaranteeing the constant phase gap. To save computing resources in CST Microwave Studio, periodic boundary condition is assigned along y axis while open condition is set to the ends of inhomogeneous array x axis to save computing resources.


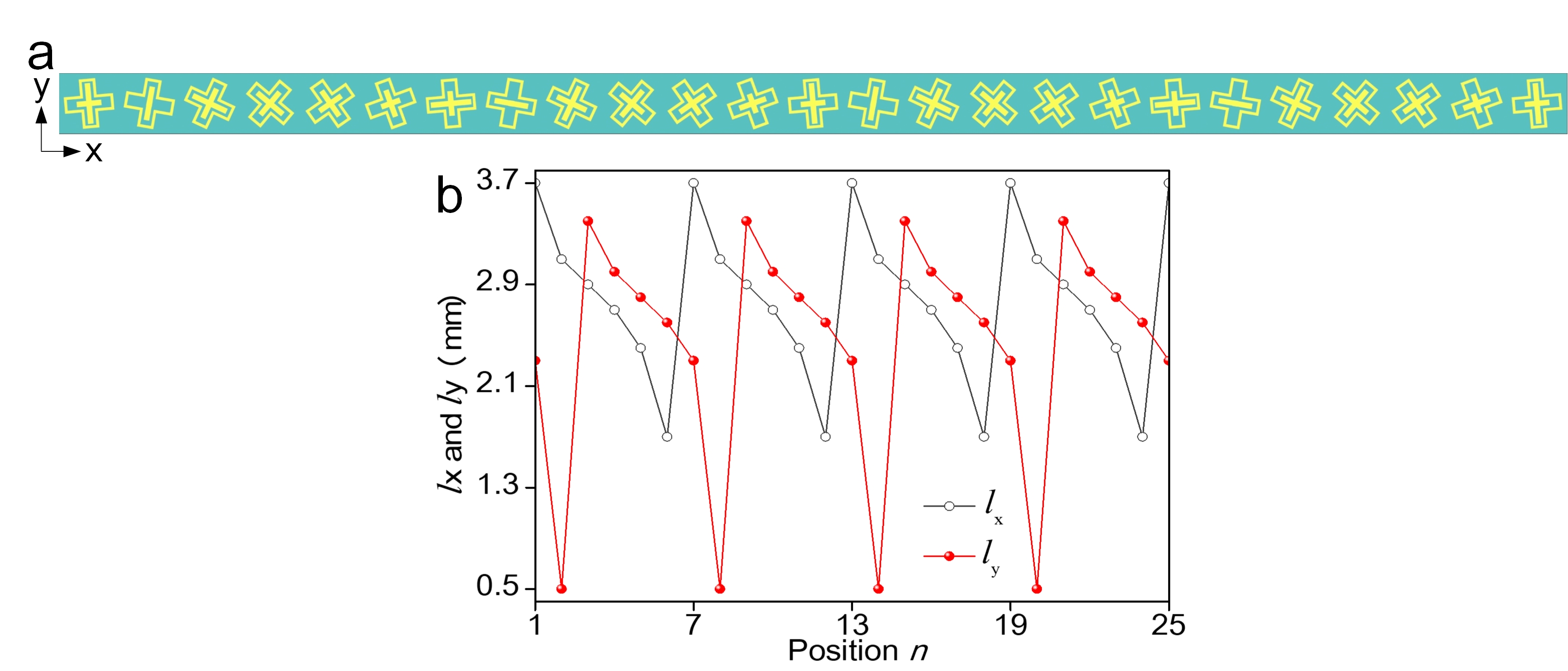


**Figure S2** (a) Layout of the meta-sheet along x axis by using (b) retrieved *l*_x_ and *l*_y_ from database.

1. **Key parameters for spin-and-momentum multiplexed retroreflector**

Figure S3 shows the retrieved key structure parameters of *l*_x_ and *l*_y_ for the final meta-sheet layout along x axis. Again, the structure parameters *l*_x_ and *l*_y_ vary point by point and the change of *l*_x_ is kept synchronously with that of *l*_y_.

**Figure S3** Retrieved *l*_x_ and *l*_y_ of the final meta-sheet layout along x axis.

1. **2D scattering patterns for spin-and-momentum multiplexed retroreflector**

Figure S4 plots the 2D cross-section scattering patterns versus elevation angles, whereas the FDTD calculations are in good consistency with the experiments. The lowest operation efficiency is measured as 86% at LL channel under -15º tilt angle incidence.


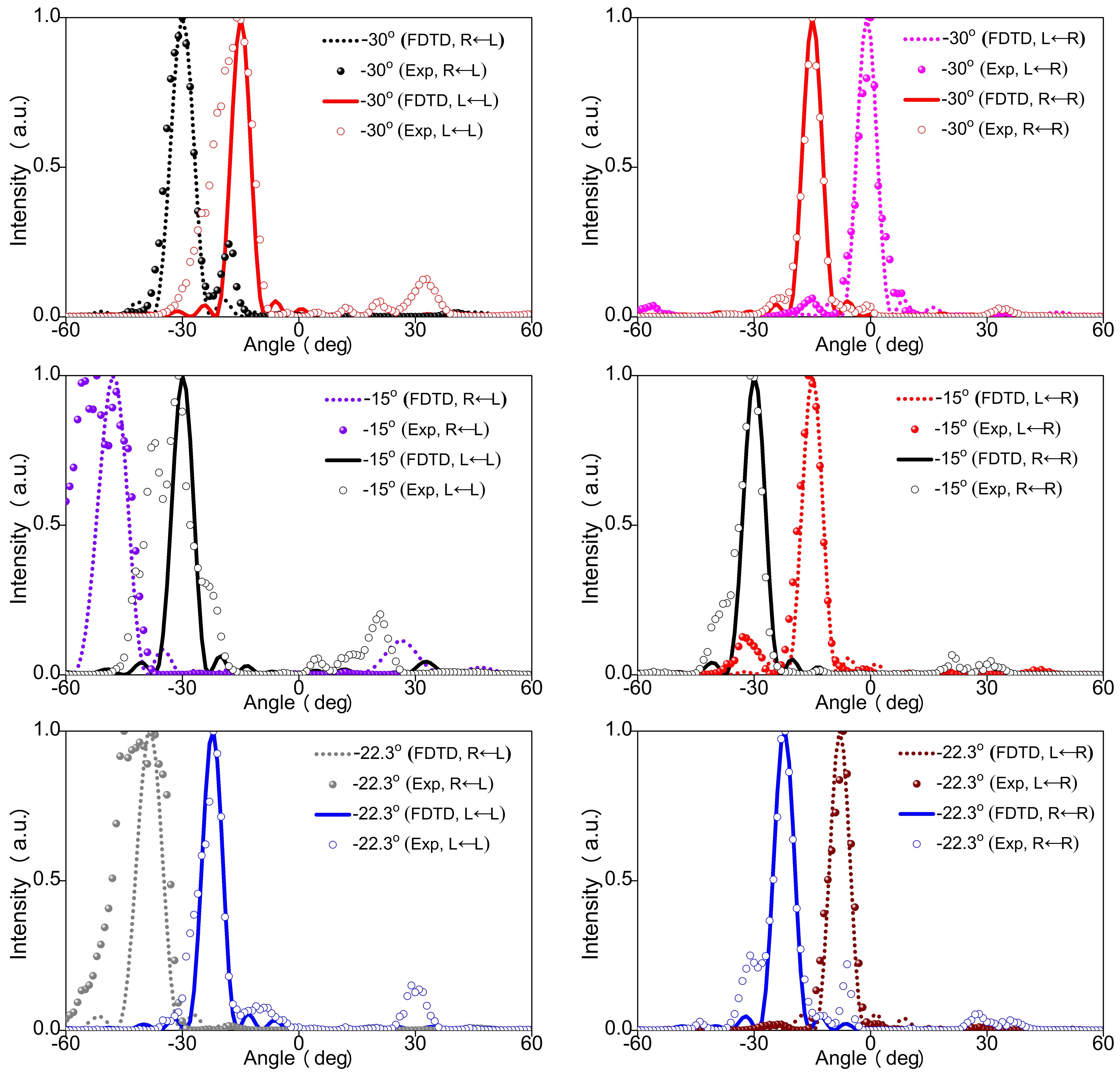


**Figure S4** Comparison of the 2D cross-section scattering patterns between FDTD calculations and experiments at 10 GHz at *θ*_i1_=-30º, *θ*_i2_=-15º, and *θ*_i3_=-22.3º by altering the CP states of input and output ends.
